# Supplementary material for: Triglyceride Glucose-Waist Circumference Better Predicts Coronary Calcium Progression Compared with Other Indices of Insulin Resistance: A Longitudinal Observational Study
Source: J Clin Med. 2020 Dec 29;10(1):92. doi: 10.3390/jcm10010092 (PMC7795085; doi:10.3390/jcm10010092)
Supplement: Supplementary file 1 [file jcm-10-00092-s001.pdf]

Supplementary materials

Table S1. Baseline clinical and biochemical characteristics according to the CAC score in the whole cohort (n = 3997).

	Total	No CAC	CAC	p
n (%)	3997	2649 (66.2)	1348 (33.7)	
Age (years)	53.2 ± 8.1	51.3 ± 7.5	56.9 ± 8.0	<0.001
Sex (male, %)	75.5	69.0	88.2	<0.001
Body mass index (kg/m^2)	24.7 ± 3.0	24.5 ± 2.9	25.3 ± 3.0	<0.001
Waist circumference (cm)	86.2 ± 8.2	85.2 ± 8.3	88.4 ± 7.7	<0.001
Systolic BP (mmHg)	119.9 ± 13.0	118.3 ± 12.7	123.2 ± 13.1	<0.001
Diastolic BP (mmHg)	76.8 ± 10.4	75.6 ± 10.4	79.2 ± 9.9	<0.001
Current smoker (%)	27.7	26.3	30.2	0.012
Moderate drinker (%)	50.4	47.3	56.5	<0.001
Physically active (%)	43.6	39.9	50.9	<0.001
Family history of diabetes (%)	24.1	24.0	24.4	0.784
Diabetes (%)	12.6	9.0	19.5	0.001
Hypertension (%)	31.0	23.8	45.2	<0.001
FPG (mg/dL)	104.5 ± 20.8	102.3 ± 18.5	108.8 ± 24.1	<0.001
HbA1c (%)	5.7 ± 0.8	5.6 ± 0.7	5.9 ± 0.9	<0.001
HbA1c (mmol/mol)	38.7 ± 8.5	37.7 ± 7.6	40.6 ± 9.9	<0.001
Total cholesterol (mg/dL)	198.0 ± 32.9	197.2 ± 32.3	199.5 ± 34.0	0.045
TG (mg/dL)	135.2 ± 82.5	130.3 ± 81.2	145.0 ± 84.6	<0.001
LDL-C (mg/dL)	124.4 ± 28.8	123.3 ± 28.1	126.5 ± 29.9	0.001
HDL-C (mg/dL)	52.6 ± 13.2	53.7 ± 13.7	50.5 ± 12.1	<0.001
Uric acid (mg/dL)	5.7 ± 1.4	5.6 ± 1.4	5.9 ± 1.4	<0.001
AST (U/L)	25 (21–31)	25 (21–31)	26 (22–32)	<0.001
ALT (U/L)	22 (17–31)	21 (16–31)	24 (18–32)	<0.001
GGT (U/L)	23 (15–39)	21 (14–37)	27 (18–42)	<0.001
hsCRP (mg/L)	0.6 (0.3–1.2)	0.5 (0.3–1.1)	0.6 (0.3–1.2)	<0.001
HOMA-IR	2.2 ± 2.2	2.0 ± 1.3	2.5 ± 3.3	<0.001
TyG index	8.7 ± 0.6	8.7 ± 0.6	8.8 ± 0.6	<0.001
TyG-BMI	216.1 ± 33.4	212.3 ± 33.1	223.5 ± 32.7	<0.001
TyG-WC	753.1 ± 100.9	738.8 ± 101.5	781.0 ± 93.8	<0.001
Baseline CAC score	0 (0–10)	0 (0–1)	33 (9–110)	<0.001

BP, blood pressure; FPG, fasting plasma glucose; HbA1c, hemoglobin A1c; TG, triglyceride; LDL-C, low-density lipoprotein cholesterol; HDL-C, high-density lipoprotein cholesterol; AST, aspartate aminotransferase; ALT, alanine aminotransferase; GGT, gamma-glutamyl transferase; hsCRP, high-sensitivity C-reactive protein; CAC, coronary artery calcification; p value showed comparison between the subjects with CAC and without CAC. Continuous variables with normal distributions are expressed as the mean ± standard deviation, whereas continuous variables with skewed distributions are expressed as the median (and interquartile range). Categorical variables are expressed as the percentage. The characteristics were compared using Student's t test for normally distributed continuous variables or Mann-Whitney U test for not normally distributed continuous variables, and the chi-squared test for categorical variables.

Table S2. The presence of CAC at baseline according to the quartiles of each parameter in the whole cohort (n = 3997).

Parameter	n	OR (95% CI)		
		Unadjusted	Model 1	Model 2
HOMA-IR				
1st quartile	1002	1.00 (Ref)	1.00 (Ref)	1.00 (Ref)
2nd quartile	999	1.14 (0.94–1.40)	1.14 (0.93–1.41)	1.08 (0.87–1.35)
3rd quartile	999	1.32 (1.09–1.59)	1.24 (1.01–1.52)	1.12 (0.90–1.40)
4th quartile	997	1.71 (1.42–2.05)	1.58 (1.29–1.94)	1.40 (1.12–1.75)
TyG				
1st quartile	998	1.00 (Ref)	1.00 (Ref)	1.00 (Ref)
2nd quartile	1000	1.60 (1.31–1.95)	1.35 (1.08–1.68)	1.23 (1.01–1.34)
3rd quartile	998	1.96 (1.62–2.38)	1.65 (1.33–2.05)	1.40 (1.10–1.78)
4th quartile	999	2.37 (1.96–2.88)	2.12 (1.71–2.63)	1.79 (1.39–2.31)
TyG-BMI				
1st quartile	999	1.00 (Ref)	1.00 (Ref)	1.00 (Ref)
2nd quartile	999	1.82 (1.49–2.22)	1.38 (1.11–1.72)	1.23 (0.97–1.56)
3rd quartile	1000	2.17 (1.78–2.65)	1.60 (1.28–2.00)	1.37 (1.07–1.76)
4th quartile	997	2.63 (2.16–3.20)	2.33 (1.87–2.91)	1.89 (1.46–2.44)
TyG-WC				
1st quartile	999	1.00 (Ref)	1.00 (Ref)	1.00 (Ref)
2nd quartile	1001	2.10 (1.71–2.58)	1.45 (1.15–1.82)	1.23 (0.96–1.58)
3rd quartile	1002	2.61 (2.13–3.19)	1.65 (1.32–2.07)	1.39 (1.07–1.80)
4th quartile	995	3.40 (2.78–4.16)	2.38 (1.90–2.99)	1.91 (1.45–2.50)

Model 1 was adjusted for age and sex. Model 2 was adjusted for the variables included in model 1, plus systolic BP, LDL-cholesterol, HDL-cholesterol, smoking, drinking, and exercise habits. Logistic regression analysis was performed to calculate the odds ratios (ORs) and 95% confidence intervals (CIs) of the subgroups for the prediction of CAC progression.

Table S3. Areas under the receiver operating characteristic curves of each parameter for CAC in the whole cohort (n = 3997).

Parameter	AUC	Standard error
HOMA-IR	0.564	0.0096
TyG	0.592	0.0093
TyG-BMI	0.599	0.0092
TyG-WC	0.622	0.0091
**Comparison ***	**Difference AUC**	***p*-Value ***
TyG-WC vs. HOMA-IR	0.058	< 0.001
TyG-WC vs. TyG	0.030	< 0.001
TyG-WC vs. TyG-BMI	0.023	< 0.001
TyG-BMI vs. HOMA-IR	0.040	< 0.001
TyG-BMI vs. TyG	0.007	1.000
TyG vs. HOMA-IR	0.014	0.028

The difference of prediction performance between the parameters were presented the ROC curve (AUC) between the models. * Comparisons were adjusted for multiple comparisons using Bonferroni correction. AUC=area under receiver operating characteristic (ROC) curves; CI=confidence interval.

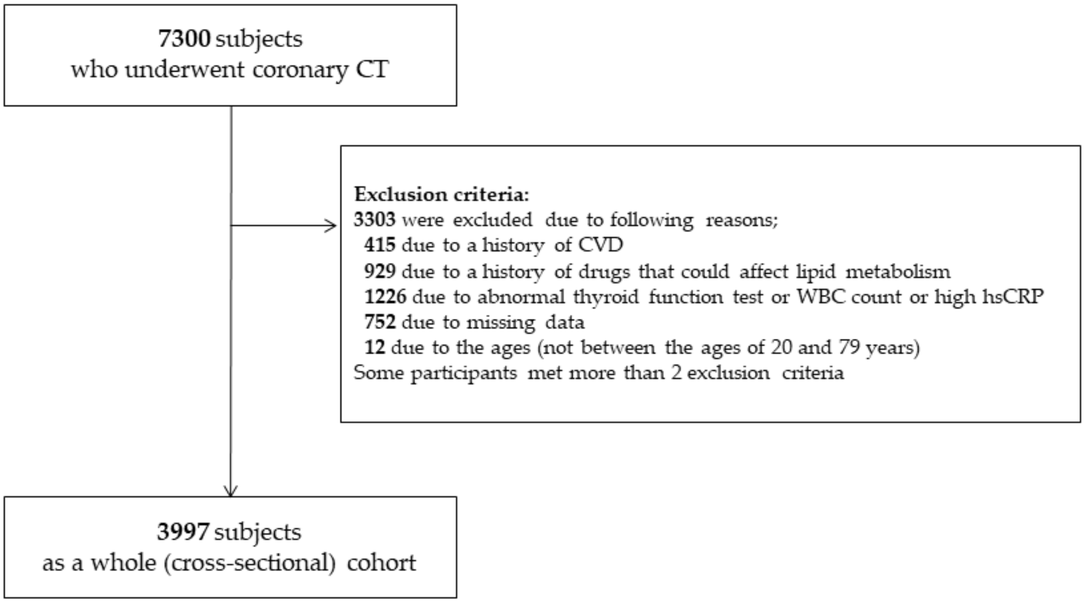

Figure S1. Study population included in cross-sectional analyses. CVD, cardiovascular disease; hsCRP, high-sensitive C-reactive protein.

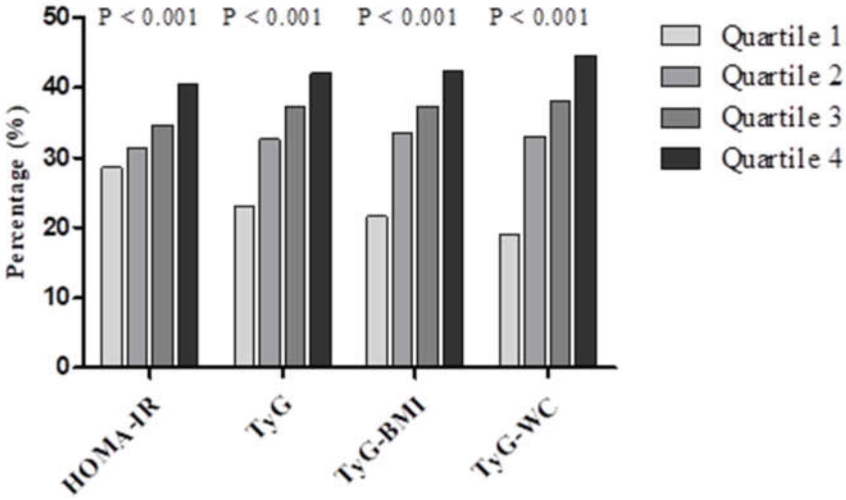

Figure S2. Proportion of the presence of CAC at baseline according to the quartiles of HOMA-IR, TyG, TyG-BMI, and TyG-WC in the whole cohort (n = 3997). The proportion of progressors was compared using the chi-squared test.

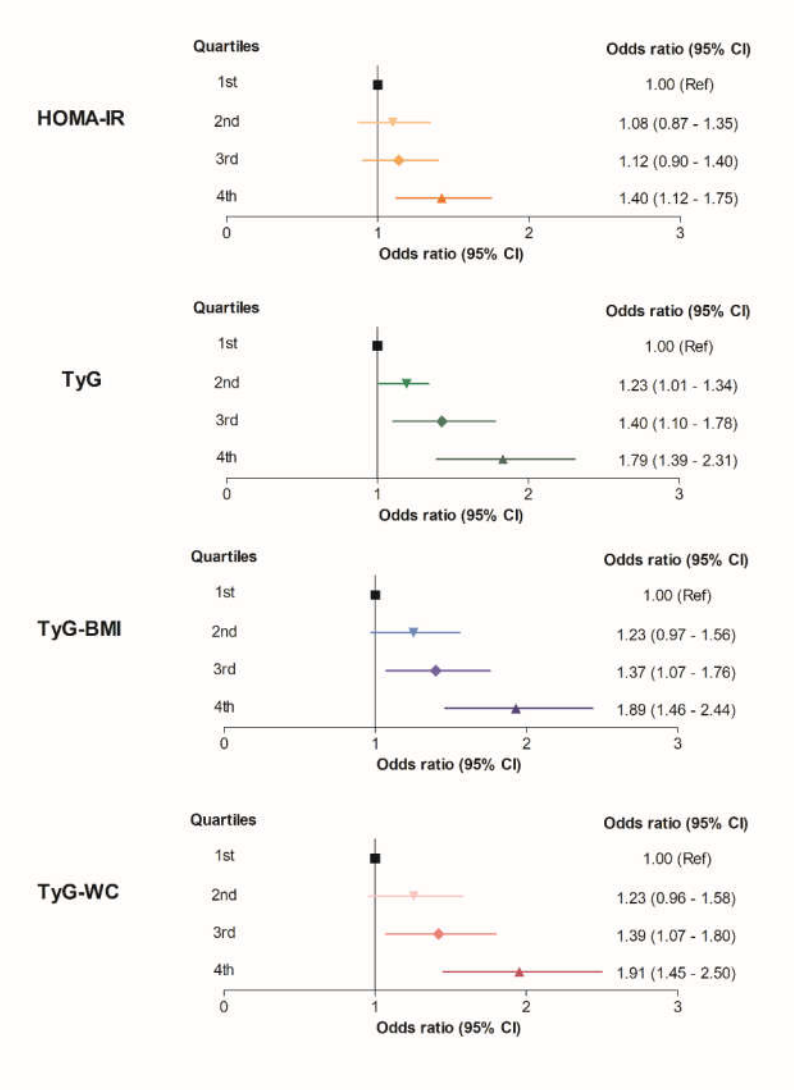

Figure S3. Summarized figure for the presence of CAC according to the quartiles of each parameter in the whole cohort (*n* = 3997). The ORs (95% CIs) are adjusted for age, sex, systolic BP, LDL-cholesterol, HDL-cholesterol, smoking, drinking, and exercise habits.

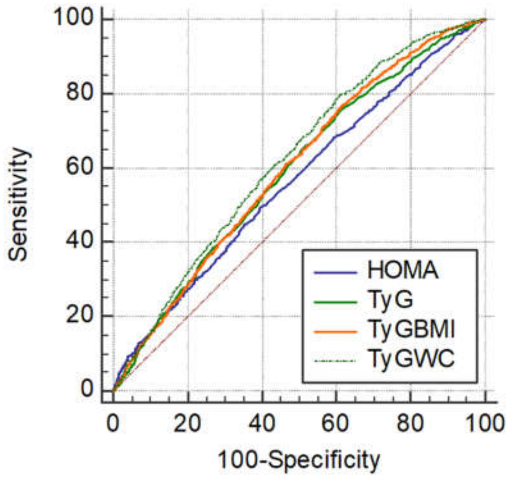

Figure S4. Receiver operating characteristic (ROC) curve of metabolic parameters for the presence of CAC in the whole cohort (n = 3997).
